# Harnessing Social Media, Influencers, and Community Engagement to Confront HIV-Related Stigma in Peru: Longitudinal Descriptive Study of the DiME Campaign

**DOI:** 10.2196/93260

**Published:** 2026-09-30

**Authors:** Alyson Nunez, Milagros Wong, Marguerite Curtis, Jhoselyn Acosta, Josue Valera, David Sanchez, Yihan Shi, Ella Chun, Kristin A Kosyluk, Jerome T Galea, Molly F Franke, Renato A Errea

**Affiliations:** 1 Department of Global Health and Social Medicine, Harvard Medical School, 641 Huntington Ave., Boston, MA, 02115, United States; 2 Programa de VIH, Socios En Salud, Javier Prado Este 492, San Isidro, Lima, Peru, 51 934 420 403; 3 Department of Mental Health Law and Policy, University of South Florida, Tampa, FL, United States; 4 School of Social Work, University of South Florida, Tampa, FL, United States

**Keywords:** youth, Peru, stereotypes, empathy, trust, education

## Abstract

**Background:**

HIV remains a public health challenge worldwide, and HIV-related stigma constitutes a major barrier to prevention, testing, and treatment efforts. Mitigating HIV-related stigma is crucial to improving outcomes for people living with HIV and reaching global HIV targets. Social media platforms and influencers are increasingly used for public health communication and may provide an opportunity to shape social norms and reduce HIV-related stigma among adolescents and young adults.

**Objective:**

This study aimed to describe the experience of implementing a Spanish-language community-engaged social marketing campaign to address HIV-related stigma among adolescents and young adults in Peru and collaborating with influencers (social media personalities with large and engaged followings) to disseminate campaign content.

**Methods:**

Based on formative community-engaged research with adolescents and young adults living with HIV, HIV advocates, and clinic providers, we developed social media content (Instagram [Meta Platforms, Inc.] and TikTok [ByteDance] platforms) that sought to address common myths and stereotypes regarding HIV, encouraged empathy for people living with HIV, and aimed to normalize conversations around testing, prevention, and treatment. Additionally, we hired 7 top Peruvian influencers (4 lifestyle influencers, 1 physician, 1 reality television star, and 1 newscaster) to create and post content from their accounts aligned with these themes. Seventy-eight Peruvian (unpaid) microinfluencers, defined as influencers with modest followings, were invited to disseminate content. All content was reviewed and approved by a community advisory board prior to posting.

**Results:**

The campaign occurred over 5 months in 2024. We created and posted 48 videos to the campaign Instagram account and 49 videos to the campaign TikTok account, yielding 1,475,918 views, 1462 comments, and 63,488 likes. The campaign generated 2206 Instagram followers and 11,800 TikTok followers, 46% of whom were in Peru. The 5 top posts created by the content creation agency and study team received 742,314 views, 1108 comments, and 50,071 likes, while the 5 top influencer posts had a total of 2,484,934 views, 1079 comments, and 129,258 likes. Forty-seven percent (684/1462) of comments on agency and study team content were positive, compared with 65% (947/1462) of comments on influencer content. Thirty unpaid microinfluencers reposted content, as did 7 additional microinfluencers not involved in the campaign. We received 512 direct messages inquiring about HIV services, requesting HIV-related information, or sharing personal experiences related to HIV.

**Conclusions:**

Social media is a useful tool for disseminating antistigma messaging. Collaboration with influencers to disseminate content was successfully implemented and expanded campaign reach.

## Introduction

HIV remains a major global public health challenge. In 2025, 41 million people worldwide were living with HIV [1]. Adolescents and young adults aged 15‐24 years represented 7.5% of this total, with 350,000 new diagnoses occurring in this population in 2025 [1]. Despite advances in HIV treatment and the recognition that people with sustained viral suppression cannot sexually transmit HIV (Undetectable=Untransmittable; U=U), HIV-related stigma continues to impede prevention, testing, and treatment efforts [2,3]. HIV-related stigma encompasses negative beliefs, attitudes, and subsequent behavior toward people living with HIV and is driven by fear, misinformation, and discrimination toward populations disproportionately affected by HIV [4-7]. It has been linked to poorer quality of life and mental health, and reduced access to care, leading to negative health consequences, including preventable suffering and death [3,8-10]. Adolescents and young adults are especially vulnerable to the effects of HIV-related stigma [11]. Mitigating this stigma is, therefore, essential to improving the health of young people living with HIV and reducing transmission.

Because HIV-related stigma is shaped by social norms and misinformation, social media, which both influences social norms and facilitates information sharing, may provide an opportunity to influence stigmatizing attitudes [12]. Social media platforms, such as Instagram (Meta) and TikTok (ByteDance), are promising tools for health promotion and education activities, particularly among adolescents and young adults, who are among the most active social media users and increasingly seek out health information using these apps [13]. In addition to their broad accessibility and popularity, social media platforms offer a mechanism for dissemination to large audiences and opportunities for audience engagement with health messaging [14-18]. The power of social media is further potentiated by social media influencers—individuals who maintain a large and engaged audience on social media [19-21]. Because influencers often cultivate trusted, parasocial relationships with their followers, they may be particularly well positioned to challenge misconceptions, model empathy, and shape social norms related to stigma [22].

Public health programs and research studies have used social media to disseminate educational health-related content and promote behaviors related to HIV prevention, HIV testing, sexual health, and other infectious diseases [23-27]. In recent years, some of these initiatives have also engaged social media influencers to increase audience reach and engagement. For example, one intervention included paid influencers to promote harm reduction measures for stigmatized bloodborne diseases such as HIV and hepatitis C, finding that their participation in the campaign was an effective strategy to promote audience reach [28]. Another study evaluated the use of influencers in a social media campaign to promote COVID-19 vaccination among the US Spanish-speaking population and found that content featuring influencers resulted in greater audience engagement compared to content that did not feature influencers [29]. While social media is increasingly gaining traction as a platform for health communication campaigns, there is limited evidence on the implementation and evaluation of influencer-led social media interventions specifically targeting HIV-related stigma, particularly in Latin America.

Peru, the target region for the social media campaign presented here, has an estimated HIV prevalence of 0.3%‐0.4%. The prevalence is notably higher in key populations such as transgender women (32.0%); gay, bisexual, or other men who have sex with men (10.0%); and female sex workers (2.4%) [30,31]. An estimated 110,000 people living with HIV reside in Peru, where there has been a recent increase in the number of new HIV diagnoses in young adults aged 18‐29 years [32]. Social media is widely used: the country of 34 million individuals includes 10.8 million Instagram users, 3.5 million of whom are aged 18‐24 years [15], and over 20 million TikTok users [16].

Given the persistent burden of HIV stigma, the increasing role of social media in health communication, and the limited evidence on social media interventions targeting HIV-related stigma specifically, we implemented “DiME” (Spanish for “Tell Me”), a Spanish-language, community-engaged social media campaign aimed at addressing HIV-related stigma among adolescents and young adults in Lima, Peru. In this paper, we describe campaign implementation on the Instagram and TikTok platforms and our experience collaborating with local social media influencers to disseminate HIV-related stigma reduction content, present campaign reach and engagement outcomes, and report lessons learned.

## Methods

### Formative Research to Inform the Campaign

We conducted formative qualitative work with a diverse group of adolescents and young adults, HIV advocates, and clinic providers to inform campaign content. Focus group and interview guides were informed by the Health Stigma and Discrimination Framework to explore key drivers and manifestations of HIV-related stigma and desired change goals (ie, what they hope would change as a result of the campaign), key messages, and content delivery strategies [6,33,34]. Participants in these focus group and interview sessions identified 2 overarching change goals: (1) reframing perceptions of HIV and living with HIV and (2) changing attitudes and actions toward people living with HIV. Key messages to achieve these goals included conveying that U=U, HIV can affect anyone, HIV is a chronic and treatable condition, and people living with HIV desire empathy, not pity.

### Social Media Content Creation

The DiME study team consisted of individuals from Peru and the United States: an infectious disease epidemiologist (MFF), a medical doctor and HIV program manager (RAE), a nurse (MW), a behavioral scientist and social worker (JTG), a stigma expert (KAK), a paid community manager and activist living with HIV (JV and DS), a social media content creator (JA), and research staff (AN, MC, YS, and EC). Most team members were bilingual in Spanish and English. We contracted a Peruvian content creation agency to create social media content that addressed HIV-related stigma, specifically for the Peruvian audience. While the agency had experience developing content related to social issues, including stigmatized infectious diseases (eg, tuberculosis), it did not have experience creating content related to HIV or stigma. To sensitize agency team members to these topics, we provided a virtual education session on clinical information and created an explanatory report including an overview of stigma, how HIV-related stigma manifests in Lima, and a summary of formative research findings [33,35]. Guided by this information, the agency created a campaign proposal using the slogan “Seamos una voz” (Be the voice)*,* which focused on dispelling stereotypes and normalizing conversations around HIV while promoting empathy, not pity. Videos debunked myths (eg, HIV and AIDS are synonymous, HIV only affects the lesbian, gay, bisexual, transgender, queer or questioning, intersex, asexual, and others (LGBTQIA+) community, HIV is transmitted through touch, and people living with HIV cannot lead “normal” lives) and educated about important concepts (eg, U=U), with each video ending with the tagline “Be the voice against prejudice” or “Be the voice against discrimination.”

The content creation agency produced a draft script of each post for review by the study team and a community advisory board made up of 15 people living with HIV, 4 of whom were HIV activists. Community advisory board members were diverse in terms of age (range: approximately 19-37 years) and sexual orientation. For each piece of content, each member of the study team and advisory board completed a brief survey to assess the appropriateness of the material and suggest improvements. The study coordinator compiled this feedback and shared it with the agency. Once produced, each piece of content underwent a second review by the study team and advisory board for final approval, or, occasionally, a decision against producing a particular piece of content. The agency filmed content around Lima featuring diverse Peruvian actors and incorporated trending music, memes, sound clips, and stickers (customizable graphic elements added to videos). Of the 35 total pieces of video content produced, 7 did not pass the approval process, and they were archived: 4 because of a misalignment between content message and campaign goals, 2 because the recorded scenes were deemed unrealistic, and 1 because actors inadvertently used stigmatizing language that could not be edited. A randomized social marketing evaluation of a subset of DiME campaign content found that brief exposure to the content, as would be experienced in a social media feed, led to detectable short-term changes in HIV-related stigma among young people recruited from the general population in Lima [36].

To achieve our accounts’ target posting frequency of twice weekly, the study team produced 21 additional videos to supplement material created by the agency. These videos provided information about sexually transmitted infection testing and treatment in Lima, addressed stigmatizing comments left on the DiME account, and shared testimonials from young adults living with HIV. The study team community manager and content creator appeared in these videos.

### Collaboration With Influencers

We contracted a Peruvian influencer management agency to identify and recruit influencers to participate in the campaign. We presented the agency with the stigma report, the campaign’s objectives, and the scope of work for influencer participation. We sought a diverse group of influencers in terms of gender, sexual orientation, and the types of content they produce. The agency proposed a diverse list of widely recognized top Peruvian influencers. The final list of paid participating influencers included 5 from this list and 2 suggested by the study team: 4 LGBTQIA+ lifestyle influencers, 1 physician, 1 reality television star, and 1 newscaster, with follower counts ranging from 117,000 to 23.8 million. Paid influencers were provided with a digital pamphlet containing information on HIV-related stigma and guidance on appropriate language to use when discussing HIV in their videos. Influencers created video scripts, which were subsequently reviewed and edited by the study team and community advisory board before filming and posting content to their platforms. Payments ranged from US $1000 to US $3200 per post. Seventy-eight microinfluencers, defined as influencers with more modest and niche followings, were identified by the management company based on perceived willingness or past campaign participation. Invited microinfluencers had 1000 to 689,000 followers and were invited to join the campaign voluntarily by reposting campaign content or by creating content based on the pamphlet shared by the study team.

### Data Collection and Analysis

We adhere to Guidelines and Checklist for the Reporting on Digital Health Implementations (iCHECK-DH; Multimedia Appendix 1) [37]. Social media indicators (ie, followers, views, likes, comments, shares, and saves) were monitored and recorded weekly by the study team during the campaign. The Instagram professional dashboard and TikTok business suite were used to extract demographic information and performance insights (ie, views and video watch time). Key performance indicators included (1) reach (total number of views), (2) interaction (total number of likes, comments, shares, and saves), and (3) engagement rate (total number of likes, comments, shares, and saves, divided by the total number of views).

Top-performing videos were considered to be those with the greatest interaction at campaign end. All direct message exchanges to the DiME account on both platforms were categorically analyzed. Following analysis, the study team purposively selected representative examples from each category for presentation in the manuscript and translated messages from Spanish to English. A sentiment analysis was performed on all user comments left on campaign content. Three study team members (AN, MC, and MFF) manually categorized comments as positive, negative, or neutral, generally and with respect to the content, using coding definitions developed by the study team based on an initial review of all comments posted (Multimedia Appendix 2). Positive comments praised the DiME initiative and expressed resonating with content, while negative comments expressed stigmatizing opinions or questioned the normalization of HIV as a chronic and treatable condition. Neutral comments included questions and answers regarding HIV, superfluous information, greetings, and ambiguous emojis. Comments were divided among the 3 reviewers (AN, MC, and MFF) for coding, with uncertainties discussed as a group to reach consensus. One study team member (AN) reviewed all final codes for consistency. To summarize sentiment polarity (ie, the balance between positive and negative sentiment), we calculated the ratio of positive to negative comments.

After completion of the campaign, all participating paid and microinfluencers were invited to complete a questionnaire asking them about their experience participating in the campaign (Multimedia Appendix 3). Responses were summarized.

### Ethical Considerations

This work was approved by the Vía Libre Bioethics Committee in Peru (CIB-8326) and by the Longwood Campus Institutional Review Board (IRB) at Harvard Medical School (IRB22-0741). Both ethical reviews covered all components of the analysis. As the IRB determined this component of the research to be exempt, influencers who completed the questionnaire were not asked to provide written consent but were informed that participation was voluntary. No compensation was provided for study participation. Prior to analysis, all data were deidentified.

### Campaign Implementation

The DiME campaign ran from July 8, 2024, to December 12, 2024, on the Instagram and TikTok social media platforms under the account name @dime.peru. These platforms were chosen for campaign implementation as they are widely used by adolescents and young adults in Peru and were considered appropriate channels for reaching the campaign’s intended audience [15,16,33]. Forty-eight videos were posted on Instagram and 49 on TikTok, with an approximate posting frequency of twice weekly. For 35% (17/48) of Instagram posts and 31% (15/49) of TikTok posts, paid boosts were used to increase reach, with a total of approximately US $564 spent across both platforms. Separately, the 7 collaborating paid influencers contributed a total of 13 videos to the campaign. Influencers posted content to their own social media accounts and directed their followers to the DiME account. Six of these videos were posted in collaboration with the DiME account, appearing simultaneously on the influencer’s page and the DiME Instagram account (collaborations were not possible on TikTok during the campaign). The DiME community manager monitored comments and direct messages, and provided timely responses to questions, misinformation, and requests for support.

## Results

### Key Performance Indicators

During the 5-month campaign, the DiME account gained 2206 followers on Instagram and 11,800 followers on TikTok. According to platform analytics obtained in December 2024 for the last month of the campaign, approximately 46% of followers had Peru-based accounts (with the next largest group of followers coming from Mexico, followed by those from neighboring countries), 64% were male, and 56% were aged 18‐34 years. Median reach per post after the first 4 weeks of the campaign was 470 on Instagram and 994 on TikTok, and increased to 4011 (IQR 1739‐13,286) and 3203 (IQR 1594‐15,100), respectively, at campaign end. Median interactions per post increased from 30 on Instagram and 54 on TikTok within the first 4 weeks, to 86 (IQR 57-206) and 110 (IQR 76-363), respectively, at campaign end. Our overall engagement rate at campaign end was 1.65% on Instagram and 6.89% on TikTok. Influencer content generated over 2.8 million views and 160,000 interactions. Metrics reported in Table 1 reflect numbers through the campaign end in December 2024.

**Table 1.** Overall key performance indicator metrics for DiME content and influencer content on the Instagram and TikTok social media platforms (July 8-December 12, 2024).

Performance indicator metrics	DiME account Instagram (n=48 posts)	DiME account TikTok (n=49 posts)	Influencer Instagram (n=6 posts)	Influencer TikTok (n=7 posts)
Total views (n), across all posts	466,215	1,009,703	361,179	2,538,973
Views per post, median (IQR)	4011 (1739‐13,286)	3203 (1594‐15,100)	46,578 (35,374-57,007)	346,592 (140,500-613,750)
Average percent of each post watched, median (IQR)	NC^a^	23.9 (19.5‐35.1)	16.3 (14.8‐26.2)	14.2^b^ (10.3‐19.8)
Total interactions (n), across all posts	7710	69,547	15,515	144,423
Interactions per post, median (IQR)	86 (57-206)	110 (76-363)	2,195 (1827‐2440)	11,489 (6866‐29,462)
Total likes (n), across all posts	6026	57,462	13,884	130,522
Likes per post, median (IQR)	67 (44-171)	96 (64-332)	2,000 (1600‐2200)	11,000 (6485‐26,500)
Total comments (n), across all posts	210	1252	356	1106
Comments per post, median (IQR)	1 (0‐4)	2 (1-7)	48 (31-72)	129 (69-199)
Total shares (n), across all posts	995	3760	585	4029
Shares per post, median (IQR)	12 (5-25)	6 (2-19)	82 (43-144)	101 (58-547)
Total saves (n), across all posts	479	7073	690	8766
Saves per post, median (IQR)	7 (3-12)	10 (5-23)	77 (61-115)	304 (224‐1844)
Engagement rate (%)	1.65	6.89	3.3-7.3^c^	1.7-14.5^d^
Direct messages (total, n)	140	372	NC	NC

a Not collected.

b Data missing for one post.

c Engagement rate on Instagram ranged from 3.3% to 7.3%.

d Engagement rate on TikTok ranged from 1.7% to 14.5%.

### Sentiment Analysis

Of the 1462 total comments received on DiME posts produced by the creation agency and study team, 684 (47%) were positive, 546 (37%) were neutral, and 232 (16%) were negative. A small percentage of comments (81/1462, 6%) conveyed antistigma messaging using hostility or negativity toward stigmatizing opinions. We classified these as positive if they endorsed the content messaging. The resulting sentiment polarity ratio (positive-to-negative) was 2.95:1, indicating nearly 3 times as many positive as negative comments. DiME account followers frequently responded to negative comments with their own messages of education:

(Regarding discrimination in the workplace)

User 1: At a private company you can hire based on internal policy. So start a company and hire people with your own policies. Period.

User 2: No internal policy is above the Constitution, and discrimination on the basis of disease is a crime.

(Regarding sero-discordant couples)

User 1: All the same, use protection. Don’t make [HIV] normal just because it’s your opinion.

User 2: You’re uninformed. If you research and ask specialists, everything they’re saying is 100% correct. Undetectable equals untransmittable.

Influencers received a total of 1462 comments on their content, 64.8% (947/1462) of which were positive. Many of their followers congratulated them for informing their audiences about HIV: “[There should be] more influencers like you who don’t just entertain but also inform.” or “I learned more in two minutes with [influencer name] than in five years of school!”

### Direct Messages

We received 512 direct messages to our accounts corresponding to the following categories: requests for HIV and sexually transmitted infection (STI) testing locations (306/512, 59.8%); questions regarding HIV testing protocols, transmission, and treatment (65/512, 12.6%); sharing of personal experiences (8/512, 1.6%); and other (133/512, 26.0%). The other classification was used for messages praising the DiME campaign, messages asking where DiME was physically located, and greetings without follow-up, among others. Messages originated from 37 localities in Lima, 16 Peruvian provinces, and 7 other Latin American countries. Using the messaging platform, the DiME community manager directed users to the public health testing facility in their locality, answered user questions, and/or provided support and encouragement. Illustrative messages are included in Table 2.

**Table 2.** Illustrative direct messages received by the DiME Instagram and TikTok social media accounts during implementation of the DiME campaign addressing HIV-related stigma among adolescents and young adults in Peru, by category.

Message category	Illustrative quotes
Requests for HIV and STI^a^ testing locations	“Hi, I saw your video about syphilis testing and I wanted to know where I could get a free test in Lima. Thank you so much.” “Hi, I saw you on TikTok. How can I get an HIV test? I’m from San Juan de Lurigancho.”
Questions regarding HIV testing protocols	“I’m not 18 yet, can I go alone as a minor? It’s just that I don’t want my parents to find out, I’d feel horrible. Seriously, thank you. I don’t know who’s behind the screen, but you’re giving me peace.” “Hi, could you help me with how long I should wait to take an HIV test? I asked, and they offered me a 4th-generation immunochromatographic test. Is that one good? I’ve been waiting three weeks, and I’m starting to have symptoms, and I don’t know if it’s because of that or the stress of not knowing.”
Questions regarding HIV transmission	“I want to know if a person on treatment and undetectable can get someone pregnant.” “Hi, I just wanted to know if someone who’s seropositive can transmit HIV. I had a seropositive partner, and at first, when they told me, I was scared, and I had this fear of getting it. Now we’re not together, and I’m scared they gave it to me. During the relationship, I got tested every three months.” “My question is if you can also get it from a tattoo (I got a tattoo and I didn’t pay attention to the needle), and I’m scared.”
Questions regarding HIV treatment or PrEP^b^	“So if I have HIV and the other person too, can we both take PREP?” “I got the diagnosis, and I’m wondering where I can start treatment as soon as possible.”
Sharing of personal experiences	“Hi, I’m new with HIV, and I’m not taking it as well as everyone else :(I don’t know if I’ll have a partner or friends or if I’ll have a good life.” “I want to file a legal complaint. How can I do that? Can you advise me? I’m HIV+ and my partner wants to tell everyone.” “I don’t know who I’m lucky enough to be talking to, but it’s really helpful to talk with someone since I’m dealing with this alone.” “Hi, this is an awesome initiative. I wish I had this information a long time ago. Someone close to me died almost three years ago, and I couldn’t help him. God willing, someday I can help someone else in the name of my brother.”
Other	“I love this, really it’s so nice to make this visible so people get a little more informed and to help them see that with treatment people can live normal lives. If you’re ever thinking about a reel or podcast, I’d love to participate.” “Where are you located? Because I’m HIV+ and I had to flee Venezuela because I couldn’t access treatment there. I’m backpacking until I can get settled and get my treatment. I wanted to know if you help in these cases.”

a STI: sexually transmitted infection.

b PrEP: preexposure prophylaxis.

### Top-Performing Content

Metrics for top-performing content are shown in Tables 3 and 4. Of the content created by the agency and study team, the top post depicted actors representing a young male same-sex couple in a sero-discordant relationship where one partner is living with HIV while the other is not. They were filmed in Lima, dispelling myths about sexual relationships while living with HIV and encouraging their peers to examine preconceived notions. This video was posted on November 8 and “went viral,” rapidly gaining substantially higher reach and engagement than typical campaign content. It received 388,276 views, 17 times the average number of views for DiME content, and 48,646 interactions, 80 times the average interactions. Other top-performing posts, as defined by the number of total interactions, dealt with rights in the workplace for young adults living with HIV, the difference between HIV and AIDS, and the stereotype that adolescents and young adults living with HIV cannot live like their peers. These videos received more than 65,000 views and 2000 interactions in total.

**Table 3.** Key performance indicator metrics for 5 top-performing videos created by the content creation agency and study team for the DiME campaign addressing HIV-related stigma among adolescents and young adults in Peru, at campaign end on December 12, 2024.

Performance indicator metrics	Same-sex sero-discordant relationship	Account of young people living with HIV not hired due to HIV	Rights of young people living with HIV in the workplace	Doctor explaining HIV versus AIDS	A person finds out neighbor is living with HIV
Views, n	388,276	95,819	69,259	116,474	72,486
Total interactions, n	48,643	3809	3540	2912	2131
Likes	39,553	3034	3055	2626	1803
Comments	500	437	75	34	62
Saves	5487	226	259	158	162
Shares	3103	112	151	94	104
Engagement rate (%)	12.53	4	5.1	2.5	2.9
Comments, n/N (%)					
Total positive	271/500 (54.2)	149/437 (34.1)	46/75 (61.3)	13/34 (38.2)	28/62 (45.2)
Total neutral	194/500 (38.8)	164/437 (37.5)	14/75 (18.7)	17/34 (50)	28/62 (45.2)
Total negative	35/500 (7)	124/437 (28.4)	15/75 (20)	4/34 (11.8)	6/62 (9.7)

**Table 4.** Key performance indicator metrics for 5 top-performing videos created by influencers for the DiME campaign addressing HIV-related stigma among adolescents and young adults in Peru, at campaign end on December 12, 2024.

Performance indicator metrics	LGBTQ+^a^ male, lifestyle influencer, teaches a “class” dispelling HIV-related myths	Male newscaster explains HIV can affect anyone	Transwoman, lifestyle influencer, debunks HIV-related stereotypes	Female reality TV star discusses empathy	Female reality TV star dispels myths about HIV
Views, n	593,000	676,000	180,000	689,342	346,592
Total interactions, n	57,149	39,598	26,083	11,489	8743
Likes	52,000	34,000	24,000	11,000	8258
Comments	413	171	282	84	129
Saves	3639	3062	1438	304	289
Shares	1097	2365	363	101	67
Engagement rate (%)	9.6	5.9	14.5	1.7	2.5
Comments, n/N (%)					
Total positive	282/413 (68.3)	82/171 (48)	196/282 (69.5)	44/84 (52.4)	59/129 (45.7)
Total neutral	116/413 (28.1)	81/171 (47.3)	69/282 (24.5)	37/84 (44)	66/129 (51.2)
Total negative	15/413 (3.6)	8 /171(4.7)	17/282 (6)	3/84 (3.6)	4/129 (3.1)

a LGBTQIA+: lesbian, gay, bisexual, transgender, queer or questioning, intersex, asexual, and others.

The 5 top-performing influencer pieces were all posted on TikTok and included videos created by lifestyle influencers in the LGBTQIA+ community, a male social media newscaster, and a female reality television celebrity. These videos all received at least 180,000 views and 8000 interactions.

Figure 1 provides a comparison between reach and interaction metrics for the top-performing videos created by the agency and study team and by influencers. Influencer numbers were greater for all categories with the exception of comments received.

**Figure 1.**
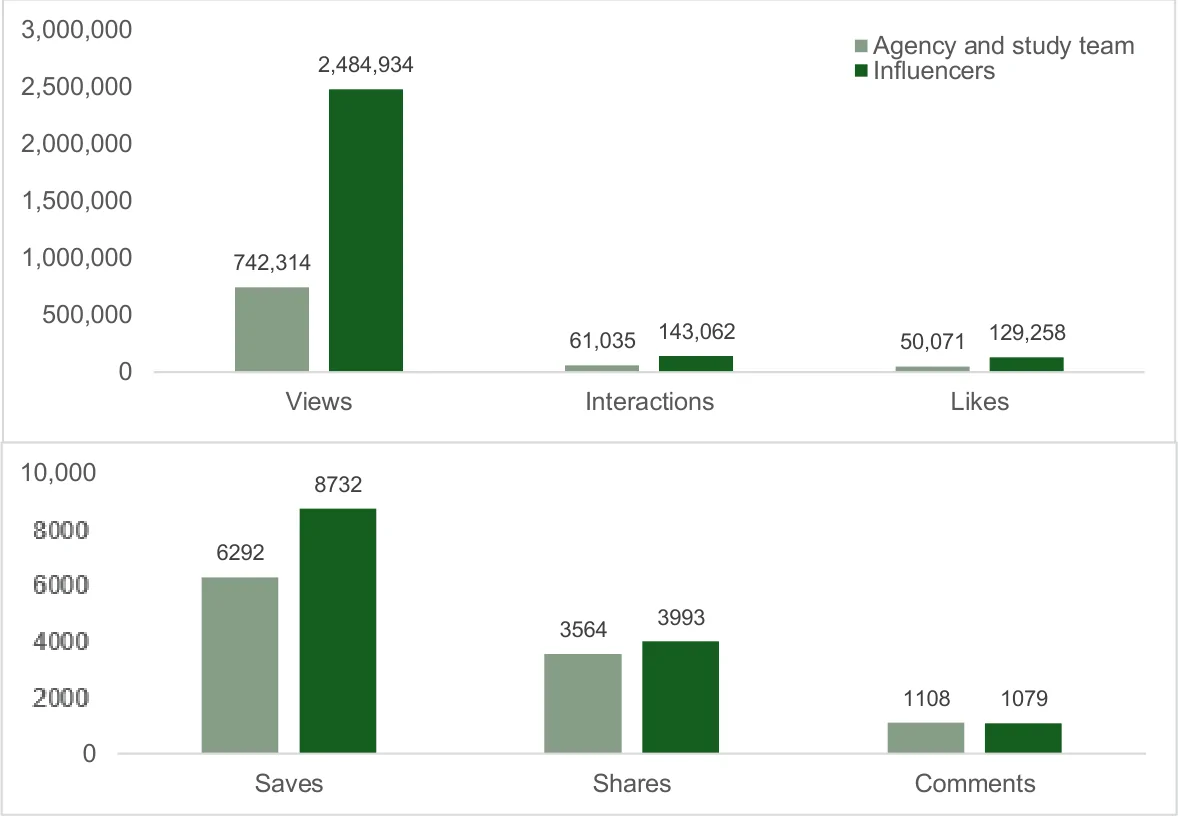
Comparison of absolute reach (views) and interaction (likes, saves, shares, and comments) for top-performing content produced by the content creation agency and study team versus influencers for the DiME campaign addressing HIV-related stigma among adolescents and young adults in Peru.

### Microinfluencers

Thirty of 78 unpaid microinfluencers (38% of total invited) shared a total of 27 and 9 posts on Instagram, created by the agency and influencers, respectively. Three influencers independently created and posted their own content about HIV-related stigma and directed their followers to the DiME account. Seven microinfluencers that were not part of the project also reposted campaign content.

### Influencer Experience Participating in the Campaign

Three paid influencers and 2 microinfluencers responded to our postcampaign survey (5/78, 6% of those invited). Three reported prior experience participating in campaigns to raise awareness about social issues, but none had experience with HIV-related campaigns. All reported accepting the invitation to participate in the campaign because it aligned with their personal values. These survey respondents reported mostly positive audience reactions to campaign content. Two indicated that participating in the campaign positively affected their image on social media, while 3 mentioned the campaign had neither a positive nor negative effect on their image. Three provided recommendations for improving communication with contracted influencers and social media followers, including considerations related to HIV-related terminology. All reported willingness to participate in future HIV-related social media campaigns.

## Discussion

### Principal Findings

Social media has become an effective and scalable tool for distributing public health information [38-40]; however, campaigns explicitly seeking to address HIV-related stigma are lacking. The DiME social media campaign sought to reduce HIV-related stigmatizing opinions and behaviors among adolescents and young adults generally, and self-stigma in adolescents and young adults living with HIV in Peru by addressing stereotypes and promoting empathy through educational messages, interviews, and testimonials. A diverse group of actors and spokespeople from the local community established the trust necessary to build a committed following, and content filmed in Lima enabled the audience to connect with the campaign messaging, identifying with the local context. Reach was substantially higher for videos created and shared by influencers compared to agency and study team videos, suggesting that influencers can feasibly support dissemination of messages intended to normalize discussions of HIV. Consistent with the Health Stigma and Discrimination Framework, the use of social media platforms and influencers provided a mechanism to address the drivers of stigma by promoting accurate information and encouraging empathy toward people living with HIV. Though this report focused on campaign implementation, subsequent analyses will examine whether exposure to the campaign reduced HIV-related stigma among young adults living with HIV.

Our experience suggests that collaborating with widely recognized paid influencers and unpaid microinfluencers on HIV-related social media campaigns is both feasible and advantageous. Working with a local influencer management agency allowed us to efficiently identify and recruit strategic personalities willing to participate. The iterative production process allowed influencers to incorporate their established communication styles and relationships with followers while ensuring that content appropriately addressed stigmatizing opinions. Microinfluencers’ sharing of DiME content further increased campaign visibility. Additionally, the fact that several unaffiliated microinfluencers also reposted content suggests that collaborating with influencers may organically extend message dissemination beyond formal campaign partnerships. These observations align with findings from previous public health campaigns that suggest influencers can positively communicate health information and increase engagement among recipient audiences [19,29,41,42]. In many parts of Latin America, conversation around HIV continues to be taboo; reaching large audiences who are not routinely exposed to HIV-related information is of utmost importance for stigma reduction [43]. We found that influencers were a highly effective means of reaching a wider and more diverse audience than could otherwise be achieved. Influencers were, in general, receptive to instruction for respectful presentation of HIV-related content, and their agreement to address these topics, despite potential negative comments, is a step toward broader public interest in HIV education. However, it is important that content creators receive training on HIV and HIV-related stigma prior to production in order to ensure that the information they share is communicated in a way that reduces stigma rather than reinforces it, while maintaining the creator’s personal style.

Establishing trust between a campaign and its audience should be a central objective of social media initiatives [44]. Previous research has shown that digital platforms can serve as trusted spaces where adolescents and young adults living with HIV have the confidence to discuss stigma and seek support [45-47]. At the end of the 5-month campaign, we had built a community on both platforms where followers shared personal experiences and asked for advice through direct messages or public comments. We believe the role of the community manager was important for obtaining this trust. This position was filled by a person living with HIV with experience in peer counseling and HIV communication, bringing lived experience and promoting authentic connection with the audience. This seemed to strengthen the campaign’s credibility and enhance the relatability of interactions with users. Preserving a balance between the desire to quickly gain followers and extend reach and cultivating human connection through slower community building is crucial to maintaining the relational dimension that enables stigma reduction. The process of trust-building took time. For the first half of our campaign period, reach and engagement were much lower than at campaign end. As such, determining the appropriate duration for public health social media campaigns should account for the time needed to cultivate an active and engaged following. Organizations should also have a transition plan or taper phase for campaign end, as once the account becomes a trusted source of information, followers expect constant engagement. We allocated resources to continue maintaining the DiME account for one year after the official campaign end, although at a reduced frequency compared with the active campaign period.

Among the lessons learned was the importance of developing content weeks in advance of the target posting date, as the nature of HIV and stigma information requires adequate time for thoughtful input regarding how content is perceived. Video first drafts were more effective than written scripts at conveying appropriate tone and visual appeal. Furthermore, language used in campaign content is critical [48]. Following the recommendation of a participating influencer, we sought to strike a balance between destigmatizing language and language understood by a lay population. For example, in Spanish, “to infect” (contagiar) is more common colloquially than the preferred term “to transmit” (transmitir). Though adolescents and young adults living with HIV may perceive the term “infect” to be derogatory, “transmit” is less commonly used and may hinder comprehension or relatability. These nuances underscore the importance of convening advisory committees of young people living with HIV and young people from the intended recipient population (ie, those not known to be living with HIV) to help strike the balance of comprehension, relatability, and respect. If possible, young adults living with HIV should accompany video recording sessions to ensure that the inadvertent use of stigmatizing language can be corrected in real time and that recorded situations mirror realistic interactions to the greatest extent possible. We were unable to incorporate this practice during the entire campaign period.

Several limitations should be considered when interpreting our implementation, reach, and engagement metrics. Though a strength of this work was our constant communication and collaboration with an advisory committee of adolescents and young adults living with HIV, this lengthened the content creation process, which led to a relatively low posting frequency of 2 videos per week. Social media algorithms reward high posting frequencies [49,50]. We chose not to create all content in advance of the campaign in order to leverage social media trends. However, the length of the content validation process meant that trends sometimes became obsolete by the time content was approved. Additionally, exact reach and engagement are difficult to assess. Adolescents and young adults may have multiple social media accounts on the same platform, inflating unique reach numbers. Stigma may hinder young people from following an HIV-related account or interacting with its content, although they have viewed it. This raises the possibility that meaningful engagement is greater than suggested by the metrics provided by campaign platforms. The occasional use of paid boosts also makes it difficult to distinguish between organic versus paid reach and engagement. Moreover, baseline differences in social media use and engagement across demographic groups may influence exposure to and interaction with campaign content; however, these factors were not considered in this study. Regarding influencer collaboration, we did not collect data on reasons for nonparticipation among microinfluencers and only 5 influencers responded to our postcampaign survey. Therefore, our understanding of influencer experiences and perspectives is limited to a small subset of participants. Future studies involving influencers could consider including an individual interview in the influencer contract. We also recognize a lack of metrics to assess the qualitative impact of users’ interactions with the DiME community manager (eg, changes in perceptions or opinions). This paves the way for future studies that measure the narrative transformation of social media users. Finally, we acknowledge that the DiME campaign was designed specifically to address stigma among social media users in Peru; however, we believe that the underlying processes used to design and implement the campaign and engage influencers are broadly generalizable.

Social media has the power to educate and cultivate empathy [51], and the potential to positively influence behavior among adolescents and young adults who are just beginning to navigate the health care system independently [52,53]. The DiME campaign used educational videos, testimonials from young people living with HIV, and influencer content to bring HIV education to this population in an accessible and engaging way. We found TikTok to be an especially promising platform for disseminating antistigma information to adolescents and young adults; the campaign gained nearly 12,000 TikTok followers in 5 months and its engagement rate of 6.89% exceeded the average 2.3% reported for the education sector on this platform [54]. However, both DiME accounts have continued to grow since the campaign ended and have garnered a place in the HIV space in Peru, with tags from other organizations’ HIV-related content. Direct messages indicate there is a critical need for messaging to reduce HIV-related discrimination. Future campaigns should consider creating content for older populations or specific groups, such as health care providers, from whom stigma and discrimination may be especially harmful [55,56].

### Conclusion

The DiME social media campaign was designed to reduce HIV-related stigma by challenging stereotypes about HIV and promoting empathy toward people living with HIV through short, engaging videos tailored to adolescents and young adults in Lima, Peru. Collaboration with popular local influencers to create and disseminate content was a novel approach in this context and served to increase campaign reach and engagement. Influencers received mostly positive feedback from their followers. To our knowledge, the DiME campaign is among the first to explicitly address HIV-related stigma via a community-based social media campaign in Latin America, adding to the growing evidence that digital platforms are promising tools for stigma reduction across languages, cultures, and health conditions.

## Supplementary material

10.2196/93260 Multimedia Appendix 1 Guidelines and Checklist for the Reporting on Digital Health Implementations (iCHECK-DH) for the DiME Social Media Campaign: Guidelines and Checklist for Reporting on Digital Health Implementations.

10.2196/93260 Multimedia Appendix 2 Sentiment Analysis Codebook.

10.2196/93260 Multimedia Appendix 3 Questionnaires exploring paid influencers’ and microinfluencers’ participation in the DiME social media campaign to reduce HIV-related stigma.

## Abbreviations

iCHECK-DH: Guidelines and Checklist for the Reporting on Digital Health Implementations
IRB: Institutional Review Board
LGBTQIA+: lesbian, gay, bisexual, transgender, queer or questioning, intersex, asexual, and others
STI: sexually transmitted infection
U=U: undetectable=untransmittable
