# Supplementary material for: Harnessing Social Media, Influencers, and Community Engagement to Confront HIV-Related Stigma in Peru: Longitudinal Descriptive Study of the DiME Campaign
Source: J Med Internet Res. 2026 Sep 30;28:e93260. doi: 10.2196/93260 (PMC13626192; doi:10.2196/93260)
Supplement: Supplementary file 3 [file jmir-v28-e93260-s003.docx]

**Supplemental Material 3: Questionnaires exploring paid influencers’ and micro-influencers’ participation in the DiME social media campaign to reduce HIV-related stigma**

**_____________________________________________________________________________________**

**Questionnaire for PAID influencers**

Thank you for taking the time to complete this questionnaire. The DiME campaign is part of a study that seeks to understand, among other things, what impact the participation of influencers has on the success of a social media campaign. For this reason your contribution is extremely valuable. All information provided will be kept confidential. The data will be analyzed and published anonymously and will not be shared outside the research team. Thank you for your time. Your answers will help us improve future campaigns to make a fairer world for everyone.

1. **What is your username on social media?**

**On Instagram: _____________________________**

**On TikTok: _______________________________**

2. **What factors influence your decision to participate or not participate in campaigns presented to you? Check all that apply.**

*If the campaign values reflect my personal values*

*If the message is relevant to my audience*

*If the campaign allows me creative freedom*

*If participating in the campaign could generate new followers for my account*

*The reputation of the brand, company or institution carrying out the campaign*

*The time involved in participation*

*The payment*

*Other, specify:*

3. **Is participating in social campaigns (campaigns that seek to raise awareness about social issues) a priority for you? Assign a value from 0 to 10, 0 meaning it is not a priority for you and 10 meaning it is a high priority for you.**
4. **Have you previously participated in social campaigns?**

*Yes (complete questions 4.1 and 4.2)*

*No*

4.1 What was the focus of the campaign(s)?

4.2 Check all the ways you have previously participated in social campaigns.

*I got paid to create my own content.*

*I made the content without payment.*

*I shared content already created.*

*Other, specify:*

5. **What motivated you to accept the invitation to participate in the DiME campaign? Check all that apply.**

*The campaign values reflect my personal values.*

*The message is relevant to my audience.*

*Participating in the campaign could generate new followers for my account.*

*The reputation of the institution carrying out the campaign, Partners in Health*

*My previous personal experiences with HIV and/or people living with HIV*

*The payment*

*Other, specify:*

6. **Was the information provided sufficient for you to create content for DiME? Check all that apply.**

*Yes, it was enough.*

*No, I would have liked more variety of topics.*

*No, I needed more detailed information.*

7. **What was your audience's reaction to the videos you created for DiME ?**

*Mostly positive*

*Neither positive nor negative*

*Mostly negative*

*I'm not sure.*

8. **How would you rate the level of engagement between the content you created for DiME and your audience?**

*The content for DiME generated more engagement than usual.*

*The content for DiME generated the same level of engagement as usual.*

*The content for DiME generated less engagement than usual.*

*I'm not sure.*

9. **Did you get any direct messages about the videos you created for DiME?**

*Yes (complete questions 9.1 and 9.2)*

*No*

9.1 Did you reply to the direct messages?

*No, none*

*Yes, some*

*Yes, most of them*

*Yes, all of them*

9.2 Did you feel prepared to answer the direct messages?

*Yes*

*No, I would have liked to have had more training and/or information on what and how to respond.*

10. **How important do you feel the information you shared has been for your audience? Assign a value from 0 to 10 indicating its importance, with 0 meaning “very irrelevant” and 10 meaning “very important.”**
11. **Did participating in the DiME campaign affect your image on social media?**

*Yes, for the better.*

*Yes, for the worse.*

*The campaign didn't affect my image.*

12. **If you had the opportunity to participate in a social campaign about HIV with us again, would you participate?**

*Yes (complete question 12.1)*

*No (complete question 12.2)*

12.1 What is the main factor that would motivate you to continue participating in social campaigns with us on social media?

*Positive reactions to my participation from my followers*

*If my participation attracts new followers to my account*

*Having the opportunity to continue raising awareness*

*Feeling a connection to the content of the campaign*

*The payment*

*Other, specify:*

12.2 What is the main factor that would prevent you from participating in social campaigns with us on social media?

*Negative reactions towards my participation from my followers*

*If I lose followers from participating in the campaign*

*Talking about these issues involves too much exposure*

*Not feeling a connection to the content of the campaign*

*The payment is not enough*

*Other, specify:*

13. **From your perspective as a content creator, how would you rate the content DiME's TikTok and Instagram accounts (**[**@** http://@dime.peru/http://@dime.peru/](http://@dime.peru)**dime.peru**[http://@dime.peru/http://@dime.peru/](http://@dime.peru)**) in terms of the following?**

|  | Very bad | Bad | Neither good nor bad | Good | Very good |
| --- | --- | --- | --- | --- | --- |
| quality of content  (e.g., graphic style, production) |  |  |  |  |  |
| frequency of content |  |  |  |  |  |
| type of content  (e.g., interviews on the street, testimonials, sketches, etc.) |  |  |  |  |  |

14. **What aspects of our content could we improve for future social campaigns about HIV?**
15. **How could we improve our work with influencers for future social campaigns about HIV? (regarding communication, logistics, materials, asks, or any other aspect)**

16. **In the future, would you be willing to participate in a social campaign about HIV for free?**

*Yes*

*No*

17. **How could we motivate influencers to participate in future social campaigns about HIV on a voluntary basis?**
18. **Is there anything else you would like to mention?**

**Questionnaire for VOLUNTEER influencers**

Thank you for taking the time to complete this questionnaire. The DiME campaign is part of a study that seeks to understand, among other things, what impact the participation of influencers has on the success of a social media campaign. For this reason your contribution is extremely valuable. All information provided will be kept confidential. The data will be analyzed and published anonymously and will not be shared outside the research team. Thank you for your time. Your answers will help us improve future campaigns to make a fairer world for everyone.

1. **What is your username on social media?**

**On Instagram:_ ____________________________**

**On TikTok: _______________________________**

2. **What factors influence your decision to participate or not participate in campaigns presented to you? Check all that apply.**

*If the campaign values reflect my personal values*

*If the message is relevant to my audience*

*If the campaign allows me creative freedom*

*If participating in the campaign could generate new followers for my account*

*The reputation of the brand, company or institution carrying out the campaign*

*The time involved in participation*

*The payment*

*Other, specify:*

3. **Is participating in social campaigns (campaigns that seek to raise awareness about social issues) a priority for you? Assign a value from 0 to 10, 0 meaning it is not a priority for you at all and 10 meaning it is a high priority for you.**
4. **Have you previously participated in social campaigns?**

*Yes (complete questions 4.1 and 4.2)*

*No*

4.1 What was the focus of the campaign(s)?

4.2 Check all the ways you have previously participated in social campaigns.

*I got paid to create my own content.*

*I made the content without payment.*

*I shared content already created.*

*Other, specify:*

5. **What motivated you to accept the invitation to participate in the DiME campaign? Check all that apply.**

*The campaign values reflect my personal values.*

*The message is relevant to my audience.*

*Participating in the campaign could generate new followers for my account.*

*The reputation of the institution carrying out the campaign, Partners in Health*

*My previous personal experiences with HIV and/or people living with HIV*

*The payment*

*Other, specify:*

6. **Did you share DiME content on your account?**

*Yes (complete question 6.1)*

*No (complete question 6.2 and skip to question 11)*

6.1 Why did you decide to share the specific content you shared?

*The topic of the content seemed important for my followers.*

*I liked the production quality of the content.*

*I felt like I connected with the content.*

*No particular reason, it was just the newest content that had come out on the DiME account when I wanted to share.*

*I chose at random.*

*Other, specify:*

6.2 Why did you decide not to share any content?

*The topics did not seem important for my followers.*

*I didn't like the quality of the content.*

*I didn't feel like I connected with the content.*

*I didn't feel comfortable sharing information about HIV on my account.*

*I forgot.*

*Other, specify:*

7. **What was your audience’s reaction to the content you shared?**

*Mostly positive*

*Neither positive nor negative*

*Mostly negative*

*I'm not sure.*

8. **How would you rate the level of engagement between the content you shared and your audience?**

*The content for DiME generated more engagement than usual.*

*The content for DiME generated the same level of engagement as usual.*

*The content for DiME generated less engagement than usual.*

*I'm not sure.*

9. **How important do you feel the information you shared has been for your audience? Assign a value from 0 to 10 indicating its importance, with 0 being “very irrelevant” and 10 being “very important.”**
10. **Did participating in the DiME campaign affect your image on social media?**

*Yes, for the better.*

*Yes, for the worse.*

*The campaign didn't affect my image.*

11. **What would have motivated you to share more DiME content with your followers?**

*If the content had been of higher quality.*

*If I had felt a connection to the DiME campaign.*

*If my followers had reacted more positively to the content I shared.*

*If you had asked me.*

*Other, specify:*

12. **Would you have joined a social media channel or a WhatsApp group where we would announce the campaign videos we had just posted so that you could repost them if you wanted to?**

*Yes*

*No*

13. **Would you be motivated to participate in a social campaign about HIV creating your own content instead of sharing content already created?**

*Yes*

*No*

14. **How could we motivate influencers to participate in future social campaigns about HIV on a voluntary basis?**
15. **Is there any way to reward the gesture of influencers who participate in a social campaign voluntarily with something other than a monetary payment?**

*Yes, please specify:____________________________________________________________*

*No*

16. **If you had the opportunity to participate in a social campaign about HIV with us again, would you participate?**

*Yes (complete question 16.1)*

*No (complete question 16.2)*

16.1 What is the main factor that would motivate you to continue participating in social campaigns with us on social media?

*Positive reactions to my participation from my followers*

*If my participation attracts new followers to my account*

*Having the opportunity to continue raising awareness*

*Feeling a connection with the content of the campaign*

*The payment*

*Other, specify:*

16.2 What is the main factor that would prevent you from participating in social campaigns with us on social media?

*Negative reactions towards my participation from my followers*

*If I lose followers from participating in the campaign*

*Talking about these issues involves too much exposure.*

*Not feeling a connection to the content of the campaign*

*The payment is not enough*

*Other, specify:*

17. **From your perspective as a content creator, how would you rate the content on DiME 's TikTok and Instagram accounts (** [**@** http://@dime.peru/http://@dime.peru/**dime.peru** http://@dime.peru/http://@dime.peru/](http://@dime.peru)**) in terms of the following?**

|  | Very bad | Bad | Neither good nor bad | Good | Very good |
| --- | --- | --- | --- | --- | --- |
| quality of content  e.g., graphic style, production) |  |  |  |  |  |
| frequency of content |  |  |  |  |  |
| type of content  (e.g., interviews on the street, testimonials, sketches, etc.) |  |  |  |  |  |

18. **What aspects of our content could we improve for future social campaigns about HIV?**
19. **How could we improve our work with influencers for future social campaigns about HIV? (regarding communication, logistics, materials, asks, or any other aspect.)**
20. **Is there anything else you would like to mention?**
